# Preoperative photobiomodulation versus nonsteroidal anti-inflammatory drugs for pain and early functional recovery after total knee arthroplasty: a randomized controlled trial

**DOI:** 10.3389/fcell.2026.1852578

**Published:** 2026-09-07

**Authors:** Juntao Lu, Lin Hao, Hongxing Guo, Yameng Xu, Jianfeng Yang, Shuai Yang, Haoran Wang, Guoqiang Zhang, Haixia Qiu, Quanbo Ji

**Affiliations:** 1 Department of Orthopedics, The Forth Center of Chinese PLA General Hospital, Beijing, China; 2 Department of Laser Medicine, Chinese PLA General Hospital, Beijing, China; 3 Department of Traditional Chinese Medicine, Xinhua Hospital Affiliated to Shanghai Jiao Tong University School of Medicine, Shanghai, China; 4 Xuan Wu Hospital Affiliated to Capital Medical University, Beijing, China

**Keywords:** nonsteroidal anti-inflammatory drugs, perioperative rehabilitation, photobiomodulation, randomized controlled trial, semiconductor laser, total knee arthroplasty

## Abstract

**Background:**

Nonsteroidal anti-inflammatory drugs (NSAIDs) are a cornerstone of multimodal analgesia after total knee arthroplasty (TKA), but gastrointestinal, renal, cardiovascular, and bleeding risks may limit their perioperative use in some older adults. This study evaluated preoperative photobiomodulation therapy (PBMT) delivered with a stationary 810 nm semiconductor laser as a non-pharmacological pre-emptive analgesic strategy compared with celecoxib.

**Methods:**

In this prospective, double-blind, double-dummy randomized controlled trial, 80 patients undergoing primary unilateral TKA were randomized 1:1 to preoperative PBMT (n = 40) or oral celecoxib (n = 40). The primary outcome was resting visual analogue scale (VAS) pain at 24 h after surgery. The non-inferiority margin was 1.0 VAS point and the assumed standard deviation for sample-size calculation was 1.5 points. Other pain time points, the Western Ontario and McMaster Universities Osteoarthritis Index (WOMAC), lower-limb swelling, early joint function, perioperative blood loss, and complications were also assessed.

**Results:**

At 24 h, resting VAS pain was 3.9 ± 1.1 with PBMT and 3.7 ± 1.0 with celecoxib (PBMT minus celecoxib mean difference, 0.20 points; 95% CI, −0.27–0.67); the upper confidence limit was below the 1.0-point margin. The 48-h WOMAC scores were 42.4 ± 5.8 and 41.8 ± 6.2, respectively (P = 0.655). PBMT was associated with smaller increases in thigh circumference on postoperative days 3 and 7, less hidden blood loss, and fewer reports of gastrointestinal discomfort.

**Conclusion:**

Preoperative 810 nm semiconductor-laser PBMT met the stated non-inferiority criterion for 24-h resting pain compared with celecoxib and was associated with favorable swelling, blood-loss, and gastrointestinal outcomes.

## Introduction

Knee osteoarthritis (KOA) is a prevalent disease among middle-aged and elderly populations, characterized by knee joint pain, swelling, and limited mobility (Price et al., 2018). As one of the most common orthopedic disorders, KOA is also a leading cause of disability in the elderly worldwide, imposing a substantial health burden on both patients and healthcare systems (Soffin and YaDeau, 2016). Statistics indicate that approximately 650 million individuals aged 40 years and above globally suffered from OA in 2020, with the incidence, prevalence, and disability rate of OA continuously rising over the past three decades—a trend also observed in China (Wainwright et al., 2020; Lewis et al., 2015). However, there is currently no curative treatment for KOA. When KOA progresses to the end stage, total knee arthroplasty (TKA) becomes the sole viable option for joint reconstruction in most patients (Bello and Holt, 2014; Bindu et al., 2020). However, the surgical procedure itself induces profound tissue trauma and local inflammatory responses, causing patients to frequently experience moderate to severe pain during the early postoperative period (Soffin and YaDeau, 2016). If not adequately managed, this severe pain can trigger sympathetic nervous system overactivation, substantially hindering early ambulation and functional exercises (Wainwright et al., 2020). This, in turn, increases the risk of complications such as deep vein thrombosis (DVT) and joint stiffness, ultimately prolonging hospital stay (Wainwright et al., 2020). Therefore, optimizing perioperative pain management is a core component of the enhanced recovery after surgery (ERAS) pathway.

Currently, multimodal analgesia is the standard protocol for perioperative pain management in TKA, within which nonsteroidal anti-inflammatory drugs (NSAIDs) play a vital anti-inflammatory and analgesic role by inhibiting cyclooxygenase (COX) activity and reducing prostaglandin synthesis (Lewis et al., 2015). However, the primary demographic for TKA comprises elderly patients who frequently present with cardiovascular and cerebrovascular comorbidities, a history of gastrointestinal ulcers, or varying degrees of renal impairment. In this vulnerable population, the routine or high-dose perioperative administration of NSAIDs (e.g., celecoxib) significantly elevates the risks of gastrointestinal bleeding, acute kidney injury, and adverse cardiovascular events (Bello and Holt, 2014; Bindu et al., 2020). Additionally, NSAIDs may inhibit platelet function, thereby increasing the risk of hidden or overt blood loss. Consequently, there is an urgent clinical need to identify an alternative or optimized regimen that effectively controls postoperative inflammation and pain while avoiding systemic adverse effects.

Photobiomodulation therapy (PBMT), also termed low-level light or laser therapy, can be delivered with red or near-infrared light and should not be attributed to a single photoacceptor (Dompe et al., 2020; Anders et al., 2015). Studies have demonstrated that near-infrared light at specific wavelengths can penetrate the skin to reach deep damaged tissues and is absorbed by cytochrome c oxidase within cellular mitochondria, thereby promoting adenosine triphosphate (ATP) production and reducing oxidative stress (Hamblin, 2017). Proposed primary mechanisms include intracellular mitochondrial photoacceptors such as cytochrome c oxidase, extracellular redox-mediated signaling such as activation of latent transforming growth factor-β1, and membrane-associated photoreceptors or ion channels, including opsins and transient receptor potential channels (Hamblin, 2018; Ponnusamy et al., 2026; Lie et al., 2022). These pathways can alter adenosine triphosphate, nitric oxide, reactive oxygen species, intracellular calcium, and downstream inflammatory signaling. Pain modulation is wavelength- and dose-dependent and may involve peripheral nociceptors and TRPV/opsin-related phototransduction rather than cytochrome c oxidase alone (Cheng et al., 2021).

Although previous studies have explored the postoperative application of laser therapy to facilitate recovery in joint arthroplasty patients, prospective clinical data regarding its use as a “pre-emptive analgesic” modality administered preoperatively to mitigate postoperative central and peripheral sensitization remain scarce. Based on the pathophysiological mechanism that TKA surgical trauma rapidly induces a severe local inflammatory response, we hypothesized that the prophylactic, localized application of a laser therapy device prior to TKA would yield anti-inflammatory and analgesic effects comparable to NSAIDs, alongside a superior safety profile.

Therefore, we designed a prospective randomized controlled trial to compare preoperative PBMT delivered by a stationary 810 nm semiconductor laser with oral celecoxib for pain control, early joint function, inflammatory outcomes, and safety after TKA. We sought to evaluate PBMT as a non-pharmacological perioperative strategy while recognizing that clinical translation depends on reproducible dosimetry and confirmation in larger multicenter studies.

## Materials and methods

### Study design and ethical standards

This was a prospective, single-center, double-blind, double-dummy randomized controlled trial. The study protocol was approved by the Medical Ethics Committee of the Fourth Medical Center of the Chinese PLA General Hospital (approval No. 2026KY070-KS001), and all participants provided written informed consent before enrollment. Because perioperative pre-emptive analgesia is an established ERAS standard for TKA, employing an absolute placebo control group without any intervention was deemed unethical. Thus, this study utilized a non-inferiority design to directly compare PBMT with a clinical standard NSAIDs intervention, employing a double-dummy design to maintain strict blinding (Boutron et al., 2010). The trial was not prospectively registered; this is reported transparently in the Abstract and considered in the limitations.

### Patient selection

A consecutive series of patients scheduled for primary unilateral TKA due to end-stage knee osteoarthritis at our joint surgery center were considered for enrollment. Inclusion criteria were: (1) age between 55 and 80 years; (2) meeting diagnostic criteria for knee osteoarthritis with failed conservative treatment; and (3) a preoperative American Society of Anesthesiologists (ASA) grade of I or II. Patients were excluded if they had: (1) an allergy to NSAIDs, history of active gastrointestinal ulcer bleeding, or severe hepatic or renal dysfunction; (2) a history of prior surgery or infection around the knee joint, or complex deformities requiring custom prostheses; (3) chronic preoperative use of opioid analgesics, glucocorticoids, or immunosuppressants; (4) contraindications to phototherapy (e.g., malignancy at the irradiation site, active tuberculosis, or severe photosensitive dermatitis); or (5) cognitive impairment precluding accurate pain assessment.

The primary endpoint for the sample-size calculation was resting VAS pain at 24 h postoperatively. The non-inferiority margin (Δ) was prospectively set at 1.0 point, corresponding to the minimal clinically important difference used in the study protocol. Assuming a standard deviation of 1.5 VAS points, a two-sided α of 0.05, 80% power, and an anticipated 15% dropout rate, 40 participants per group (80 total) were required.

### Randomization, blinding, and interventions

Eligible participants were randomly assigned 1:1 to the phototherapy or celecoxib group using a computer-generated random-number table. An independent research assistant who was not involved in treatment or outcome assessment placed allocations in sealed opaque envelopes. To support the double-dummy design, participants in the phototherapy group received active PBMT plus placebo capsules, whereas participants in the celecoxib group received active celecoxib plus sham PBMT. Placebo capsules were prepared in identical-appearing packaging and administered on the same schedule as celecoxib. Sham PBMT was delivered in the same treatment room, with the participant positioned identically, the applicator placed approximately 15 cm above the affected knee for 30 min, and the device display and operating procedure kept consistent, but therapeutic laser emission was disabled. The staff member operating the treatment and the ward nurse administering capsules were not involved in outcome assessment. Participants, surgeons, anesthesiologists, ward nurses, outcome assessors, and statisticians were blinded to allocation.

The phototherapy group received treatment with a stationary, mains-powered semiconductor laser (810 nm wavelength, continuous-wave mode) (Bahrami et al., 2022; Huang et al., 2022). The device had two radiators, but only one radiator was activated for single-channel output. The nominal output power was 400 mW. The activated radiator produced a rectangular treatment field of 120 mm × 210 mm (252 cm^2^), corresponding to an irradiance of approximately 1.59 mW/cm^2^ at the treatment field. The applicator was positioned perpendicular to the skin surface approximately 15 cm above the affected knee, and non-contact vertical irradiation covered the upper surface of the knee joint. Each session was delivered continuously for 30 min without subdivision into multiple contact points; at 400 mW, the emitted energy was 720 J per session and the nominal fluence was 2.86 J/cm^2^ per session. Two preoperative sessions were administered at 24 h and 1 h before surgery, giving a total emitted energy of 1440 J and a cumulative nominal fluence of 5.71 J/cm^2^. The celecoxib group received 200 mg orally twice daily beginning 24 h before surgery; the final dose was administered 1 h before surgery with no more than 50 mL of water under ward-nurse supervision (Lubis et al., 2018; Xu et al., 2019a).

### Perioperative management

All surgeries were performed by the same senior joint surgery team using a standard technique: a midline knee incision and a medial parapatellar approach. A cemented posterior-stabilized (PS) prosthesis was used in all cases, without patellar resurfacing, and a tourniquet was routinely applied intraoperatively.

All patients received standard general anesthesia combined with an ultrasound-guided adductor canal block (20 mL of 0.375% ropivacaine). Postoperatively, all patients were equipped with an intravenous patient-controlled analgesia (PCIA) pump (sufentanil combined with tropisetron hydrochloride). If patients reported a resting VAS score of ≥4 points with inadequate relief from the PCIA, subcutaneous morphine hydrochloride (5 mg) was administered as rescue analgesia (Bajjuri et al., 2024).

### Postoperative rehabilitation

Both groups received the same standardized postoperative rehabilitation program, delivered by the same rehabilitation team and independent of group allocation. Rehabilitation began on the day of surgery after recovery from anesthesia and hemodynamic stabilization, with ankle-pump exercises, quadriceps and gluteal isometric contractions, active or active-assisted knee flexion-extension, and bed-to-chair transfer training as tolerated. From postoperative day 1, patients were encouraged to bear weight as tolerated with a walker or crutches and received supervised physiotherapy twice daily during hospitalization, including gait training, progressive knee range-of-motion exercises, straight-leg raises, quadriceps strengthening, balance training, and stair practice before discharge when appropriate (Jette et al., 2020). Discharge instructions included the same home program for both groups, with daily walking progression and repeated knee flexion-extension and strengthening exercises.

### Blood loss calculation

Total and hidden blood loss were calculated using the Gross approach (Gross, 1983). Patient blood volume (PBV) was estimated with the sex-specific Nadler formula (Nadler et al., 1962): PBV (L) = 0.3669 × height (Wainwright et al., 2020) (m^3^) + 0.03219 × weight (kg) + 0.6041 for men, and PBV (L) = 0.3561 × height (Wainwright et al., 2020) (m^3^) + 0.03308 × weight (kg) + 0.1833 for women. Hematocrit was measured within 24 h before surgery and on the morning of postoperative day 3. Total blood loss was calculated as PBV × (Hctpre - Hctpost)/Hctave, where Hctave = (Hctpre + Hctpost)/2. Visible blood loss was the intraoperative measured blood loss, and hidden blood loss was calculated as total blood loss minus visible blood loss plus transfused red-cell volume, if any. No participant received an allogeneic red-cell transfusion during the study period; therefore, transfused red-cell volume was zero for all calculations.

### Functional outcomes

The primary endpoint was resting VAS pain (0–10) at 24 h after surgery. Resting and dynamic VAS pain at the other prespecified time points and WOMAC total scores were additional outcomes. The validated Chinese WOMAC Likert 3.1 format was used; it contains 24 items covering pain, stiffness, and physical function, each scored from 0 (none) to four (extreme), giving a total score range of 0–96, with higher scores indicating worse symptoms and function (Symonds et al., 2015). Other secondary outcomes included thigh circumference (measured 10 cm proximal to the superior patellar pole), active knee range of motion, time to first out-of-bed ambulation, HSS score, blood loss, and complications.

### Statistical analysis

Data were analyzed using SPSS version 26.0. Normally distributed continuous variables are presented as mean ± standard deviation and were compared with independent-samples t tests; skewed variables were compared with Mann-Whitney U tests, and categorical variables with chi-square or exact tests as appropriate. Repeated outcomes were analyzed with repeated-measures analysis of variance. For the primary non-inferiority comparison, the PBMT-minus-celecoxib mean difference in 24-h resting VAS pain and its two-sided 95% confidence interval were calculated. Non-inferiority was concluded if the upper confidence limit was below Δ = 1.0 point. All 80 randomized participants completed the assigned intervention and follow-up, so the intention-to-treat and per-protocol populations were identical. Statistical significance for superiority analyses was set at P < 0.05.

## Results

### Patient enrollment and baseline characteristics

**FIGURE 1:**
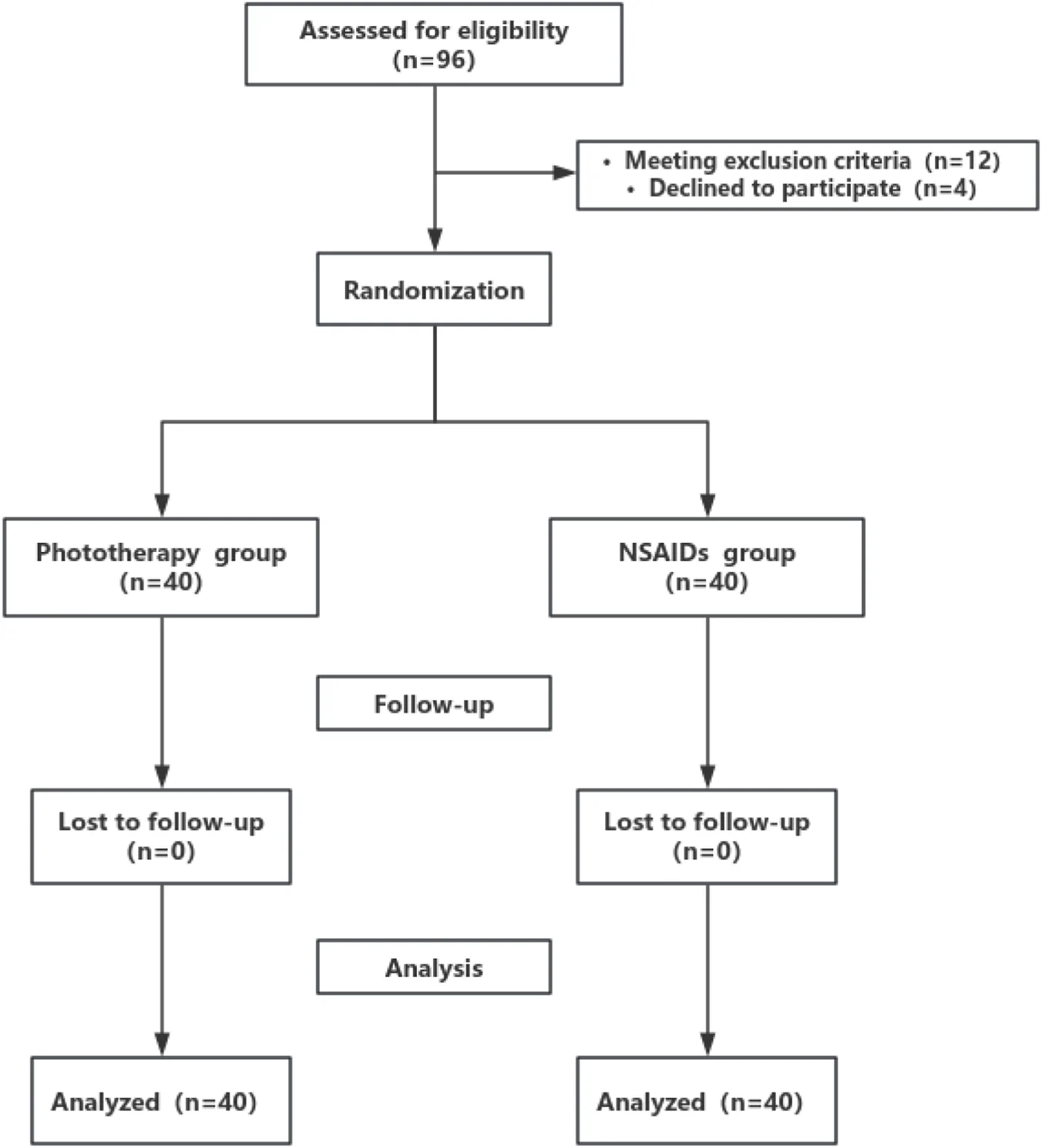
Flow of participants through enrollment, randomization, follow-up, and analysis.

Initially, 96 patients scheduled for primary unilateral TKA were assessed for eligibility, and 16 patients were excluded (12 did not meet the inclusion criteria, and 4 declined to participate). Ultimately, 80 patients were successfully randomized into the phototherapy group (n = 40) or the NSAIDs group (n = 40) (Figure 1). All patients completed the intervention protocol and follow-up. The two groups did not differ significantly with respect to baseline characteristics (Table 1).

**TABLE 1:** Baseline characteristics of patients.

Characteristic	Phototherapy group (N = 40)	NSAIDs group (N = 40)	P value
Demographics			
Age (yr), mean ± SD	66.8 ± 6.2	67.3 ± 5.9	0.705
Sex (Male/Female), n (%)	15 (37.5)/25 (62.5)	17 (42.5)/23 (57.5)	0.648
BMI (kg/m^2^), mean ± SD	26.1 ± 3.4	26.5 ± 3.1	0.582
ASA grade (I/II), n	14/26	11/29	0.496
Preoperative comorbidities, n (%)			
Hypertension	16 (40.0)	18 (45.0)	0.648
Type 2 diabetes mellitus	8 (20.0)	9 (22.5)	0.785
Preoperative clinical assessments			
Resting VAS pain score	5.5 ± 1.2	5.7 ± 1.1	0.437
Knee ROM (°)	91.5 ± 10.4	90.8 ± 11.0	0.765
Hemoglobin (g/L)	133.4 ± 11.5	134.8 ± 12.1	0.591
Serum CRP (mg/L)	4.3 ± 1.6	4.6 ± 1.8	0.433

BMI, body mass index; ASA, american society of anesthesiologists; VAS, visual analogue scale; ROM, range of motion; CRP, C-reactive protein. Continuous variables were analyzed using the independent-samples t-test; categorical variables were analyzed using the chi-square test.

### Postoperative pain and early functional recovery

At 24 h after surgery, mean resting VAS pain was 3.9 ± 1.1 in the PBMT group and 3.7 ± 1.0 in the celecoxib group. The PBMT-minus-celecoxib mean difference was 0.20 points (95% CI, −0.27–0.67); because the upper confidence limit was below the 1.0-point margin, the stated non-inferiority criterion was met. Resting and dynamic pain scores decreased in both groups, with no statistically significant between-group differences at the reported time points. The 48-h WOMAC total scores were 42.4 ± 5.8 and 41.8 ± 6.2, respectively (P = 0.655). Rescue morphine was required by 5/40 (12.5%) and 4/40 (10.0%) participants on the day of surgery and by 2/40 (5.0%) in each group on postoperative day 1 (Table 2). Because each rescue administration consisted of 5 mg subcutaneous morphine, recorded rescue-morphine exposure through postoperative day 1 was 35 mg in the PBMT group and 30 mg in the celecoxib group. Using the conventional parenteral-to-oral morphine conversion factor of 3, these doses corresponded to 105 and 90 oral morphine milligram equivalents (MME), or 2.63 and 2.25 MME per randomized participant, respectively. The PCIA regimen was standardized across groups; patient-level pump activation logs were not used for efficacy comparison.

**TABLE 2:** Postoperative pain, WOMAC scores, and rescue analgesia.

Outcome measure	Phototherapy group (N = 40)	NSAIDs group (N = 40)	P value
Resting VAS pain score			
Postoperative day 1	3.9 ± 1.1	3.7 ± 1.0	0.395
Postoperative day 2	3.0 ± 0.9	2.9 ± 0.8	0.597
Postoperative day 3	2.2 ± 0.7	2.0 ± 0.8	0.237
Postoperative 2 weeks	1.4 ± 0.6	1.3 ± 0.5	0.420
WOMAC score (total)			
Preoperative baseline	58.2 ± 6.5	57.9 ± 7.1	0.843
Postoperative 48 h	42.4 ± 5.8	41.8 ± 6.2	0.655
Postoperative day 7	31.5 ± 4.2	32.1 ± 4.5	0.540
Patients requiring rescue analgesia, n (%)			
Day of surgery	5 (12.5)	4 (10.0)	0.723
Postoperative day 1	2 (5.0)	2 (5.0)	1.000
Rescue morphine exposure through postoperative day 1, oral MME, total (mean per randomized participant)	105 (2.63)	90 (2.25)	Not tested

VAS, visual analogue scale; WOMAC, Western Ontario and McMaster Universities Osteoarthritis Index; MME, morphine milligram equivalents; WOMAC, was scored with the validated Chinese Likert 3.1 format (24 items, total range 0–96), with higher scores indicating worse pain, stiffness, and functional limitation. For resting VAS, pain on postoperative day 1, the PBMT-minus-celecoxib mean difference was 0.20 points (95% CI, −0.27–0.67); the non-inferiority margin was 1.0 point. Rescue morphine exposure was converted to oral MME, using a parenteral morphine conversion factor of 3.

#### Inflammation, swelling, and early joint function

Postoperatively, serum CRP levels increased substantially from baseline in both groups. However, on postoperative day 3, the peak CRP level in the phototherapy group trended slightly lower than that in the NSAIDs group (38.5 ± 18.4 mg/L vs. 45.2 ± 22.1 mg/L, P = 0.141). The phototherapy group demonstrated a significant advantage in controlling local tissue edema. On postoperative day 3, the increase in thigh circumference was significantly smaller in the phototherapy group than in the NSAIDs group (+3.2 ± 1.1 cm vs. +4.1 ± 1.4 cm, P = 0.021). Benefiting from milder local swelling, the time to first independent out-of-bed standing was slightly earlier in the phototherapy group (20.5 ± 5.2 h vs. 22.1 ± 6.0 h, P = 0.201). By the 4-week follow-up, both groups had recovered well in terms of knee ROM and Hospital for Special Surgery (HSS) scores, with no significant intergroup differences (P > 0.05) (Table 3).

**TABLE 3:** Comparison of postoperative early joint function outcomes.

Outcome measure	Phototherapy group (N = 40)	NSAIDs group (N = 40)	P value
Increase in thigh circumference (cm)			
Postoperative day 1	2.2 ± 0.9	2.5 ± 1.0	0.160
Postoperative day 3	3.2 ± 1.1	4.1 ± 1.4	0.021*
Postoperative day 7	1.8 ± 0.8	2.4 ± 1.1	0.007*
Time to first out-of-bed ambulation (hr)	20.5 ± 5.2	22.1 ± 6.0	0.201
Knee ROM (°)			
Postoperative 2 weeks	104.5 ± 8.6	103.2 ± 9.1	0.513
Postoperative 4 weeks	115.2 ± 6.4	114.8 ± 6.8	0.784
HSS knee clinical score			
Preoperative baseline	56.4 ± 8.5	55.9 ± 9.0	0.795
Postoperative 4 weeks	88.6 ± 5.2	87.9 ± 5.5	0.556

ROM, range of motion; HSS, Hospital for Special Surgery. * Significant, P < 0.05.

### Perioperative blood loss and complications

The phototherapy group had lower observed total and hidden blood loss than the celecoxib group and fewer reports of gastrointestinal discomfort or acid reflux (1/40 vs. 8/40, P = 0.013). These findings are associations from secondary outcomes and should not be interpreted as proof that PBMT directly altered platelet or coagulation function, which was not measured. The incidence of postoperative nausea and vomiting was similar between groups, and no deep-vein thrombosis, deep periprosthetic infection, or massive gastrointestinal bleeding was reported during follow-up (Table 4).

**TABLE 4:** Comparison of perioperative blood loss and complication rates.

Indicator/Complication	Phototherapy group (N = 40)	NSAIDs group (N = 40)	P value
Total blood loss (mL)	425.6 ± 150.2	510.4 ± 180.5	0.024*
Intraoperative overt blood loss (mL)	120.4 ± 40.5	124.8 ± 45.3	0.648
Postoperative hidden blood loss (mL)	305.2 ± 118.4	385.6 ± 145.2	0.009*
Complications, n (%)			
Gastrointestinal discomfort/acid reflux	1 (2.5)	8 (20.0)	0.013*
PONV	3 (7.5)	5 (12.5)	0.456
Deep vein thrombosis (DVT)	0	0	1.000
Delayed wound healing	0	1 (2.5)	1.000

* Significant, P < 0.05. PBV, was estimated with the sex-specific Nadler formula, and total blood loss was calculated as PBV × (Hctpre - Hctpost)/Hctave using hematocrit values measured within 24 h before surgery and on postoperative day 3. Hidden blood loss was total blood loss minus visible blood loss plus transfused red-cell volume, if any. No participant received allogeneic red-cell transfusion. PONV, postoperative nausea and vomiting.

## Discussion

TKA is an effective treatment for end-stage knee diseases; however, severe postoperative pain and robust local inflammatory responses can significantly impede early rehabilitation, prolong hospital stays, and increase the risk of postoperative complications (Price et al., 2018; Soffin and YaDeau, 2016; Wainwright et al., 2020; Lewis et al., 2015; Kehlet, 2015). Driven by the ERAS concept, multimodal analgesia has become the gold standard for perioperative TKA management, with NSAIDs serving as a foundational component (Xu et al., 2019a; Fillingham et al., 2020; Gaffney et al., 2017; Berend et al., 2004). Nevertheless, patients undergoing TKA are generally elderly and frequently present with cardiovascular, gastrointestinal, or hepatorenal comorbidities. Chronic or high-dose NSAID use not only significantly increases the risk of gastrointestinal bleeding and acute kidney injury but may also impair platelet function, exacerbating perioperative blood loss (Bello and Holt, 2014; Bindu et al., 2020; Bajjuri et al., 2024; Lanas and Chan, 2017; Harirforoosh et al., 2013). These pharmacological and perioperative analgesia considerations are consistent with prior evidence-based reviews of NSAID use and multimodal pain-management strategies (Ong et al., 2007; Crofford, 2013; Rainsford, 2007; Memtsoudis et al., 2018). In this randomized trial, the upper bound of the 95% confidence interval for the PBMT-minus-celecoxib difference in 24-h resting VAS pain was below the stated 1.0-point non-inferiority margin. The observed pain trajectories and early WOMAC scores were otherwise similar between groups. PBMT was also associated with less thigh swelling, total and hidden blood loss, and gastrointestinal discomfort (Langella et al., 2018; Stausholm et al., 2019; Vassao and Laakso, 2022; Hegedus et al., 2009); because these were secondary outcomes in a modest single-center sample, they should be interpreted as hypothesis-generating rather than definitive evidence of clinical superiority.

The pain findings are consistent with prior studies of PBMT after arthroplasty and in knee osteoarthritis (Langella et al., 2018; Stausholm et al., 2019; Vassao and Laakso, 2022; Hegedus et al., 2009; Kheshie et al., 2014; Alghadir et al., 2014). Unlike previous studies that primarily administered interventions postoperatively, our study innovatively advanced the intervention timing to 24 h and 1 h preoperatively. The theoretical rationale for this “pre-emptive analgesic” strategy is to block the transmission of peripheral nociceptive signals to the central nervous system before the onset of surgical trauma and noxious stimuli, thereby effectively preventing the development of central sensitization (Lubis et al., 2018; Wang et al., 2024; Woolf, 2011; Xu et al., 2019b). But the present clinical data cannot establish a specific neural or cellular mechanism. Furthermore, a single parameter set cannot define the optimal PBMT dose: wavelength, irradiance, fluence, treatment duration, tissue geometry, and timing may interact and may produce non-linear or biphasic responses (Zein et al., 2018; Hamblin, 2018; Cheng et al., 2021).

The smaller increase in thigh circumference after PBMT may be clinically relevant because swelling can restrict mobility and early knee function (Pua, 2015; Loyd et al., 2019; Holm et al., 2010; Mizner et al., 2005). Mechanistically, PBMT should not be described solely as near-infrared activation of mitochondrial cytochrome c oxidase. Both red and near-infrared wavelengths can engage intracellular chromophores, extracellular redox-sensitive pathways, and membrane-associated opsin/TRP signaling, with downstream effects on nitric oxide, calcium, reactive oxygen species, and inflammatory mediators (Hamblin, 2018; Ponnusamy et al., 2026; Lie et al., 2022; Cheng et al., 2021). Earlier PBMT studies also described mitochondrial light absorption, nitric-oxide release, NF-κB/ROS signaling, inflammatory-cell modulation, and muscle-recovery effects (Karu, 1999; Passarella and Karu, 2014; Chung et al., 2012; Lohr et al., 2009; Bjordal et al., 2006; Chen et al., 2011; Aimbire et al., 2006; Alves et al., 2013; Borsa et al., 2013). Moriyama et al. demonstrated wavelength-dependent modulation of iNOS expression and inflammatory-cell infiltration in an experimental mouse-knee inflammation model, underscoring that wavelength and dose can materially alter the biological response (Moriyama et al., 2005). Because the present trial did not measure synovial or tissue biomarkers, these pathways remain biologically plausible explanations rather than mechanisms demonstrated by our data.

The lower frequency of gastrointestinal symptoms in the PBMT group is consistent with avoidance of perioperative celecoxib exposure, but the trial was not powered for uncommon adverse events. Gastrointestinal and cardiovascular risks of NSAIDs and celecoxib have been described in prior safety literature (Scarpignato et al., 2015; Moore et al., 2007; Solomon et al., 2017). Likewise, the lower observed blood loss cannot be attributed directly to platelet or coagulation effects because neither pathway was measured. Blood loss after TKA is influenced by surgical technique, tourniquet use, tranexamic acid, fluid balance, and the calculation method. Hidden-blood-loss and blood-management studies after arthroplasty further support explicit calculation and transfusion accounting, while fibrinogen biology links tissue injury, coagulation, and inflammation (Gross, 1983; Sehat et al., 2004; Dhawan et al., 2017; Noticewala et al., 2012; Carling et al., 2015; Zhang et al., 2020; Xie et al., 2017; Luyendyk et al., 2019). Replication with prospectively standardized blood-management protocols and explicit transfusion accounting is therefore required before these secondary findings can guide practice.

This study has several limitations. It was conducted at one center with 80 participants and was not powered to detect uncommon serious complications. The trial was not prospectively registered, which should be considered when interpreting the strength and transparency of the non-inferiority claim. Follow-up ended at 4 weeks, so chronic postsurgical pain and long-term functional or implant outcomes were not assessed (Wylde et al., 2017). The study evaluated one PBMT parameter set and therefore cannot establish an optimal dose-response relationship or justify direct routine translation to other devices, treatment geometries, or clinical populations. The patient-level PCIA pump activation log was not analyzed, so opioid-equivalent reporting was limited to the recorded rescue morphine exposure. Finally, no synovial-fluid, histological, or molecular analyses were performed, so the cellular mechanisms discussed above remain hypotheses grounded in prior literature (Mountziaris et al., 2011).

## Conclusion

In this single-center randomized trial, preoperative PBMT delivered with a stationary 810 nm semiconductor laser met the stated non-inferiority criterion for 24-h resting pain compared with celecoxib and was associated with less postoperative swelling, blood loss, and gastrointestinal discomfort. These secondary findings require confirmation, and the present study does not establish an optimal PBMT dose or readiness for routine clinical substitution of NSAIDs. Registered multicenter trials with complete dosimetry, standardized rehabilitation, patient-level opioid-equivalent reporting, and longer follow-up are needed before broader clinical translation.

## Data Availability

The original contributions presented in the study are included in the article/supplementary material, further inquiries can be directed to the corresponding authors.
